# Risk factors and antimicrobial resistance profiles of *Pseudomonas putida* infection in Central China, 2010–2017

**DOI:** 10.1097/MD.0000000000017812

**Published:** 2019-11-01

**Authors:** Genmei Tan, Yang Xi, Peihong Yuan, Ziyong Sun, Daofeng Yang

**Affiliations:** a Department of Infectious Disease; b Department of Orthopedics; c Department of Clinical Laboratory, Tongji Hospital, Tongji Medical College, Huazhong University of Science and Technology, Wuhan, Hubei, China.

**Keywords:** Antimicrobial resistance, clinical features, *Pseudomonas putida*, risk factors

## Abstract

The aim of this study was to analyze the risk factors, clinical features, and antimicrobial resistance of *Pseudomonas putida (P putida*) isolated from Tongji Hospital in Wuhan, China.

The data of 44 patients with *P putida* infections were retrospectively reviewed in this study. All cases of *P putida* strains were detected by the clinical laboratory of Tongji Hospital in the period of January 2010 to December 2017. Antimicrobial susceptibility testing was conducted using Kirby-Bauer method.

Forty-four effective strains of *P putida* were isolated, including 32 inpatients and 12 outpatients. The 32 inpatients cases were obtained from various departments, which were urosurgery wards (n = 5, 15.6%), pediatrics wards (n = 4, 12.5%), hepatic surgery wards (n = 4, 12.5%), among others. The isolates had been discovered from urine specimens (28.2%), blood specimens (21.9%), sputum specimens (12.5%), and so on. Twenty-five patients had histories of catheterization before the isolation of *P putida*. Twenty-four patients were in immunocompromised states, 5 patients had undergone surgery, catheterization and were taking immunosuppressive therapy simultaneously. Polymicrobial infections were found in some *P putida* cases, especially *Stenotrophomonas maltophilia*, *Pseudomonas aeruginosa*, and *Escherichia coli*. All the patients had treated by antimicrobial before culture. Multi-drug-resistant strains were detected in 75% of *P putida* isolates. The *P putida* strains were resistant to trimethoprim/sulfamethoxazole (97.7%), aztreonam (88.6%), minocyline (74.3%), ticarcillin/clavulanic acid (72.7%), and sensitive to amikacin (86.4%), imipenem (62.8%), gentamicin (56.8%).

Catheterization or other invasive procedures, immunocompromised states, and underlying diseases increased the risks of *P putida* infections. Moreover, the *P putida* strains were highly resistant to trimethoprim/sulfamethoxazole, aztreonam, minocyline, ticarcillin/clavulanic acid.

**HIGHLIGHTS**

- Local data on *Pseudomonas putida* infection in China are limited. This study contains the largest number of *Pseudomonas putida* infection cases in the literature until now.
- Antimicrobial resistance profiles of *Pseudomonas putida* infection has changed with time.
- This study concludes the clinical features and risk factors of *Pseudomonas putida* infection.

## 1 Introduction

*Pseudomonas putida*, a specialized aerobic organism of the fluorescent group of *Pseudomonas* species, is a pathogenic bacterium of fish which can also colonize the human throat.^[1–3]^ It can be widely found in inanimate hospital surfaces and moist environments because of its strong tolerance to hard living conditions.^[3,4]^ Moreover, *P putida* can cause infections in hospitals because of its various infection and transmission routes.^[5]^ However, compared with other *Pseudomonas* species, it was previously thought to be of low pathogenicity. Previous studies have shown that *P putida* was sensitive to most antimicrobial agents, so clinical cases caused by *P putida* were uncommon.^[6]^

In recent years, the isolation rate of *P putida* has been rising yearly, and the emergence of multi-drug-resistant (MDR) strains, even extensively drug-resistant strains (XDR) of *P putida* had became a cause for concern.^[7,8]^ At present, there are few articles in the literature—most case reports are related to the infections and antimicrobial resistance of *P putida*, making it difficult for us to analyze the clinical features and the prevalence of *P putida* resistance. To further understand the infection profiles of *P putida* and its resistance to common antimicrobials in recent years, we reviewed 44 cases of *P putida* infected during January 2010 to December 2017 in a large teaching hospital in central China.

## 2 Materials and methods

### 2.1 Clinical specimen and information collection

Forty-four cases of *P putida*-infected patients (including outpatients) were identified during January 2010 to December 2017 through a review of the clinical microbiology laboratory records in Tongji Hospital, Huazhong University of Science and Technology, a comprehensive healthcare organization also served as education facility for both Department of Healthcare and Education in Wuhan, China. Then, the inpatients’ data, including the age, sex, distribution of wards, underlying diseases, comorbidities, indwelling devices, co-pathogens, drug resistance, and administering of antimicrobial before culture, were collected from the electronic medical records of Tongji Hospital. Finally, we analyzed the clinical features, risk factors, and antimicrobial resistance of the data and finally draw conclusions in the following parts.

### 2.2 Bacterial identification and the antimicrobial susceptibility testing

The bacterial culture procedures were followed by the “National Clinical Laboratory Operation Regulations” (Version 3) and the kit instructions. In addition, we used the Vitek II Compact Automated System (BioMé roués, France) and the Bruner Maldi-Tof MS System Mass Spectrometer (Bruker GmbH, Germany) to identify the *P putida* strains. Antimicrobial susceptibility was determined for all isolates by the disk diffusion testing (no inhibition zone). *Pseudomonas aeruginosa* ATCC27853 and *Escherichia coli* ATCC25922 were used as reference strains for quality control. Inhibition zone diameters were measured and interpreted according to Clinical and Laboratory Standards Institute guidelines criteria. The final results showed sensitive (S), intermediate (I), and resistant (R). The antimicrobial agents involved were as follows: trimethoprim/sulfamethoxazole, ciprofloxacin, gentamicin, amikacin, imipenem, ceftazidime, aztreonam, piperacillin, cefoperazone/sulbactam, levofloxacin, cefepime, piperacillin/tazobactam, meropenem, minocyline, ticarcillin/clavulanic acid, cefoperazone, tobramycin. The susceptibility disc was provided by OX-OID Company. All reagents were qualified before use.

## 3 Results

### 3.1 Specimen source and the distributing of *P putida*

A total of 44 effective strains of *P putida* were isolated from 32 inpatients and 12 outpatients. The clinical data of the 12 outpatients were not available because they have no records in the electronic medical system. Only the first bacterium episode for each patient was included in the analysis. The clinical data of the 32 inpatients were listed below (Table 1).

**Table 1:**
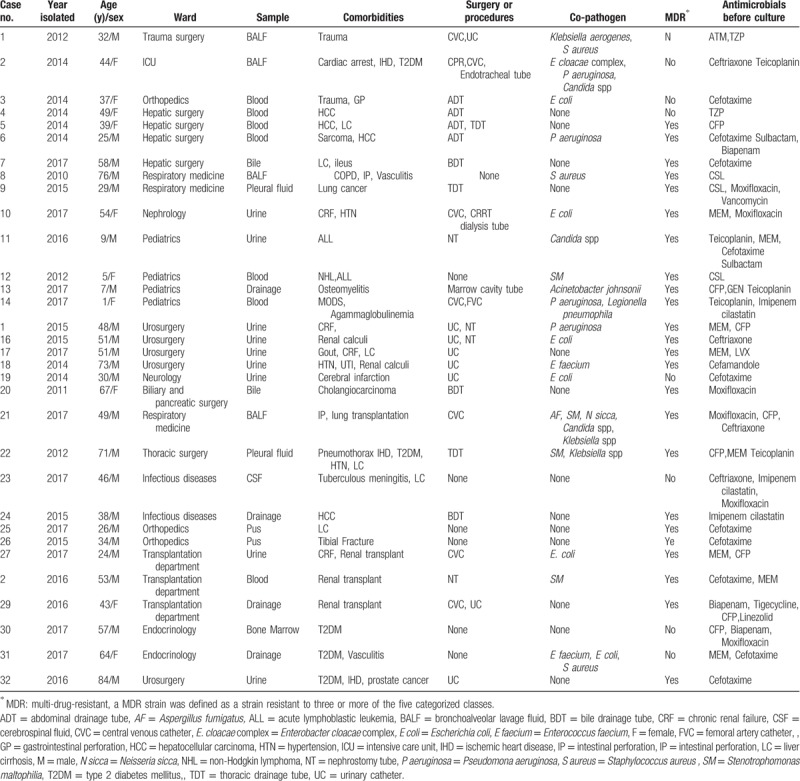
Clinical data of 32 inpatients with *P putida* infection.

**Figure 1:**
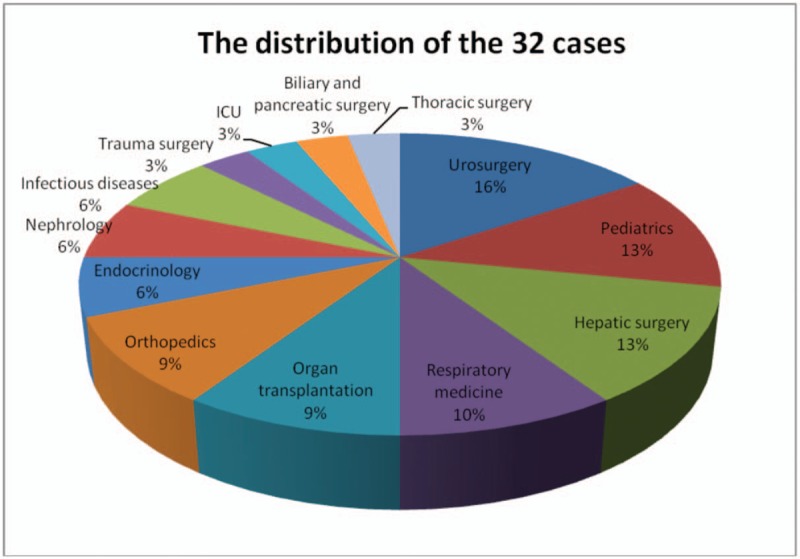
The distribution of the 32 cases.

**Figure 2:**
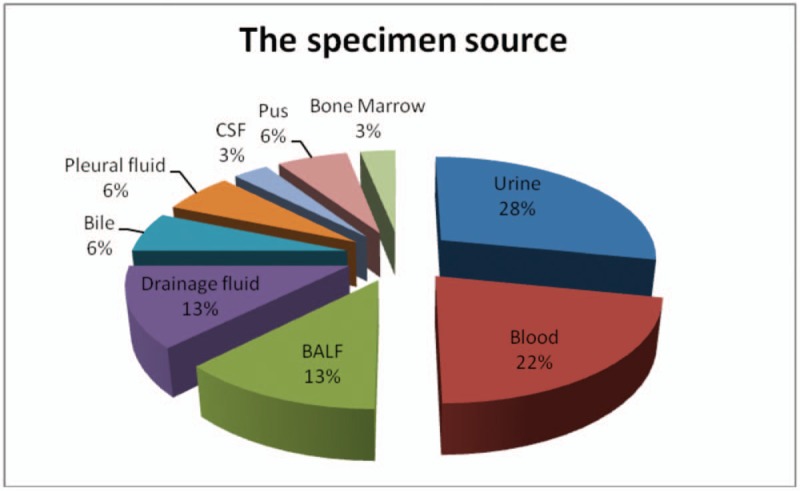
The specimen source.

Among the 32 inpatients cases, 5 (15.6%) were isolated between January 2010 and December 2013 and 27 (84.4%) between January 2014 and December 2017. The majority were male (n = 22, 68.8%), and many of them were above 40 years’ old (n = 18, 56.3%). Additionally, the distribution of the 32 cases according to hospital wards was as follows: urosurgery (5, 15.6%), pediatrics (4, 12.5%), hepatic surgery (4, 12.5%), respiratory medicine (3, 9.4%), organ transplantation (3, 9.4%), orthopedics (3, 9.4%), endocrinology (2, 6.3%), infectious diseases (2, 6.3%) and others (6,18.8%) (Fig. 1). Culture-positive samples included urine (9, 28.1%), blood (7, 21.9%), sputum (4, 12.5%), drainage fluid (4, 12.5%) and samples from other sources (8, 25.0%) (Fig. 2).

### 3.2 Risk factors

Twenty-five patients (78.1%) had indwelling catheters, such as biliary drainage tubes, urinary catheters, or femoral venous catheters. Among them, 7 patients had histories of surgery, 8 patients took immunosuppressants, 4 patients had a history of trauma, and 4 patients received radiochemotherapy recently. Besides, 24 (75%) patients had been admitted for various underlying diseases, including myocardial infarction, hypertension, chronic renal failure, liver cirrhosis, acute lymphoblastic leukemia, hepatocellular carcinoma, diabetes mellitus, among others. Meanwhile, 5 patients (15.6%) had undergone surgery, catheterization, and immunosuppressive therapy simultaneously.

All the patients had received antimicrobials before culture. The most common antimicrobial agent was cephalosporin (26, 81.3%), followed by carbapenem (14, 43.8%), quinolones (7, 21.9%), and teicoplanin (5, 15.6%). Others (18, 56.3%) had been administered ≥2 antimicrobial agents simultaneously, including piperacillin/tazobactam, tigecycline, amikacin, and linezolid, among others. Moreover, 24 of the 32 (75%) hospitalized patients were infected with MDR strains of *P putida*.

### 3.3 Clinical manifestations

Polymicrobial infections were frequent (19, 59.4%). The common superinfection microbes comprised of *E coli* (6, 18.8%), *Stenotrophomonas maltophilia* (4, 12.5%) and *P aeruginosa* (4, 12.5%). Other pathogens including *S aureus*, *Aspergillus fumigatus, Candida* spp, and *E cloacae* complex. *Klebsiella* spp and *Legionella pneumophila* were also detected in some cases. In addition, 6 patients (18.8%) detected with >2 pathogens.

The most common clinical manifestation of *P putida* infection was fever. Patients also showed frequent urination, burning with urination, abdominal pain, diarrhea, tachypnoea, cough, headaches, and among others. An increased white blood cell count, the elevated levels of interleukin-6, procalcitonin, and C-reactive protein were mainly found in laboratory analysis. After an effective treatment, there were no deaths in our study.

### 3.4 Prevalence of *P. putida* resistance

The 44 strains of *P putida* (including 12 strains from outpatients) were tested for susceptibility to 17 commonly used antimicrobials. The *P putida* strains were resistant to trimethoprim/sulfamethoxazole (97.7%), aztreonam (88.6%), minocyline(74.3%), ticarcillin/clavulanic acid (72.7%), cefoperazone/sulbactam (54.5%), ciprofloxacin (54.5%), and cefoperazone (52.3%), and were sensitive to amikacin (86.4%), imipenem (62.8%), gentamicin (56.8%), and meropenem (45.5%). The results on susceptibility tests were shown in Table 2.

**Table 2:**
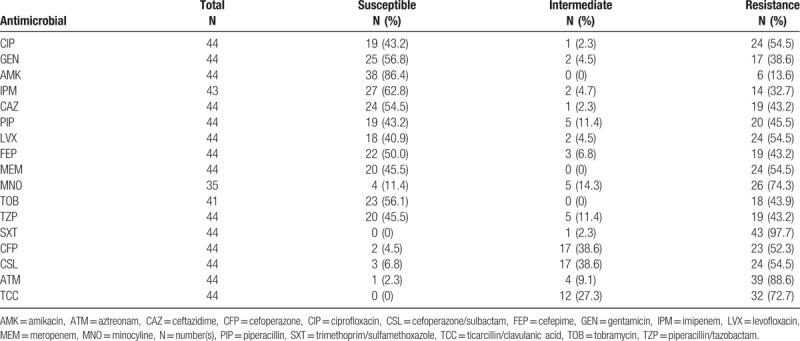
Antimicrobial resistance of *P putida* strains to 17 common antimicrobials.

## 4 Discussion

The cases of human infections of *P putida* had been first reported from blood during 1980 and 1985 in 15 patients with cancer.^[9,10]^ After that, patients with pneumonia, catheter-related bloodstream infections, acute cholecystitis, cholangitis, tonsillitis, thrombophlebitis, skin, and soft tissue infections have been ever reported to be infected with *P putida*,^[5,11–13]^ Most of studies have shown that *P putida*, which acted as an opportunistic pathogen,^[14,15]^ often infected patients who were in an immunocompromised state and had a poor physical condition.^[10,16–18]^ Besides, *P putida* was ever considered as a bacterium with low toxicity, weak pathogenicity, showed a high susceptibility to many antimicrobials, and finally had a good prognosis. However, recent studies indicated that the mortality rate in *P putida-*infected patients with underlying disease was high (40%),^[11,19]^ which has gradually aroused clinician's concern. Despite the fact that this organism can cause healthcare-related infections, clinical data on *P putida* infections are relatively lacking owing to the rarity. To date, the literature about *P putida*-related infections were mostly case reports, and few large case series were found, thus making it hard to analyze the clinical characteristics and the prevalence of *P putida* resistance. In this study, we collected 44 cases of *P putida* infections, which might contained the largest number of *P putida* infection cases in the literature until now. Among the 32 inpatient cases, most were elderly or children, 24 inpatients (75%) were in immunocompromised states (including tumor, cirrhosis, taking immunosuppressive agents after transplantation, and so on), 25 inpatients (78.1%) had a history of catheterization or catheter insertion(especially indwelling urinary catheter) before the isolation of *P putida*, which had the same trends with previous studies.^[10,11,19,20]^ Besides, one of the other main ways of *P putida* invasion was through bloodstream infection. Our results showed that the bloodstream infection of *P putida* mainly occurred in patients with organ transplants, hematologic diseases, and tumors. In these patients, the therapeutic procedures were required for primary diseases, as well as the poor conditions of the patients significantly increased the risks of *P putida* infection. As a result, implementing aseptic precaution, enhancing the immunity of patients, and blocking the infection route (device removal) were necessary for reducing infection risk of *P putida* and shortening the duration of hospitalization during the treatment of susceptible individuals or application of invasive procedures.

As for detection methods of *P putida,* at early time, the classic strategy for bacterial identification was based initially on fast and simple tests, and performed by using either commercial kits such as miniaturized biochemical tests (API analysis) or automated systems. After that, the use of protein profiles obtained by Matrix-Assisted Laser Desorption Lonization Time of Flight Mass Spectrometry directly from colonies was successfully proposed and developed gradually. Although molecular biology developed in recent years enabled rapid bacterial identification using polymerase chain reaction (PCR), which was one of the most sensitive test, the cost and workload requirements currently preclude their routine use. In our research, we used the Vitek II Compact Automated System and the Bruker Maldi-Tof MS System Mass Spectrometer to identify the *P putida* strains which identification results were reliable.

In previous reports, clinical isolates of *P putida* showed low resistance to most antimicrobials. For example, Sader et al, reported that from 1997 to 2003, the resistant rates of *P putida* to levofloxacin and ciprofloxacin were 20.2% and 21.7%, respectively.^[21]^ Afterward, *P putida* isolates were usually reported increasing resistance to common antimicrobials, including carbapenem. Our study showed that its resistance rates to trimethoprim/sulfamethoxazole were up to 90%, and quinolones and cefoperazone/sulbactam were >50%. However, *P putida* has a high susceptible rate to amikacin (86.4%), higher than the data from Sader et al (79.8%). This difference is probably a result that clinicians in China often choose cephalosporins or fluoroquinolones as the first choice rather than aminoglycosides in clinical works, when it comes to the infection of *P putida* or other unknown bacteria. Sulfonamides, a competitive inhibitors of dihydropteroate synthase, as reported in the study,^[22]^ was widely applied in the clinical and agricultural fields, causing the extensive resistance to various bacteria (included *P putida* certainly). In this study, the rate of resistance of *P putida* strains to trimethoprim/sulfamethoxazole was >97%, which was consistent with the previous studies.^[1,22]^ In addition, it can be seen from the collected cases that *P putida* maintained a higher sensitivity to imipenem and amikacin compared with other antimicrobials. Thus, imipenem and amikacin can be used as references for clinical practice. Furthermore, in our study, 24 of the 32 (75%) hospitalized patients were infected with MDR strains of *P putida*, and the MDR strains showed a broadly resistance trend to 17 common antimicrobial. Among the 24 inpatients, 2 children with acute lymphoblastic leukemia developed resistance to all antimicrobials, which increased the difficulty of treatment, duration of hospital stay, economic burden, and the morbidity of patients. Therefore, when it came to pathogenic infections, a rational selection of antimicrobial agents was critical for patients. Besides, multiantimicrobial combinations and effective surveillance of resistance will reduce the generation of drug-resistant strains and finally improve the prognosis of patients.^[20]^

There are some limitations associated with our study. First, organism identification was identified by using an automated system and was not performed by genotypic-based methods. Along similar lines, demonstration of antimicrobial resistance genes was not performed, and characterization of resistance profiles was carried out based upon disk diffusion data only. However, according to Jacquier et al, 8 of 9 *P putida* isolates identified by 16S rRNA gene sequencing were confirmed accurately by using the Vitek II Compact Automated System and there was no misidentification.^[23]^ The common microbials mentioned in this article, such as *P putida*, were not difficult to identify. It must be kept in mind that this manuscript was not intended to provide a detailed microbiological analysis but rather was meant to be a broad survey of the isolation patterns and susceptibility profiles of *P putida*. Second, this study was retrospective and had a limited number of cases. Because of its retrospective nature, it was not possible to confirm the pathogenic role of all of the identified isolates, and we failed to exclude factors associated with other pathogens in polymicrobial infection. Moreover, we could not fully avoid contaminants of *P. putida* from other sources, such as the environment and endogenous sources. Furthermore, this study was a single-center; the results obtained from this study were not generalizable enough. Ideally, a multicenter study is essential and meaningful for future research to determine whether these results represent a local or global phenomenon. Despite these limitations, this study provided risk factors, clinical characteristics, and antimicrobial susceptibility of *P putida* infection.

## 5 Conclusion

This study demonstrated that *P putida* infections, mostly presented as polymicrobial infections, were predisposed to patients with underlying diseases, immunocompromised state, a history of catheterization, or other invasive procedures. The *P putida* strains had showed high resistance rates to most antimicrobials, such as trimethoprim/sulfamethoxazole, aztreonam, minocyline, ticarcillin/clavulanicacid, cefoperazone/ sulbactam, ciprofloxacin, cefoperazone, and so on.

## Acknowledgments

The authors are grateful for their technical assistance.

## Author contributions

**Conceptualization:** daofeng yang.

**Data curation:** Genmei Tan.

**Formal analysis:** Genmei Tan.

**Funding acquisition:** daofeng yang.

**Investigation:** Genmei Tan.

**Methodology:** Genmei Tan.

**Project administration:** Genmei Tan.

**Resources:** Ziyong Sun.

**Software:** Yang Xi.

**Supervision:** Yang Xi, Ziyong Sun, daofeng yang.

**Validation:** Peihong Yuan.

**Visualization:** Peihong Yuan.

**Writing – original draft:** Genmei Tan.

**Writing – review & editing:** Genmei Tan.

## References

[1] Docquier JD Riccio ML Mugnaioli C IMP-12, a new plasmid-encoded metallo-beta-lactamase from a *Pseudomonas putida* clinical isolate. Antimicrob Agents Chemother 2003;47:1522–8. 12709317 10.1128/AAC.47.5.1522-1528.2003 PMC153319

[2] Laffite A Kilunga PI Kayembe JM Hospital effluents are one of several sources of metal, antibiotic resistance genes, and bacterial markers disseminated in sub-saharan urban rivers. Front Microbiol 2016;7:1128. 27499749 10.3389/fmicb.2016.01128 PMC4956658

[3] Devarajan N Kohler T Sivalingam P Antibiotic resistant Pseudomonas spp. in the aquatic environment: a prevalence study under tropical and temperate climate conditions. Water Res 2017;115:256–65. 28284092 10.1016/j.watres.2017.02.058

[4] Rodriguez A Escobar S Gomez E Behavior of several Pseudomonas putida strains growth under different agitation and oxygen supply conditions. Biotechnol Prog 2018;34:900–9. 29603901 10.1002/btpr.2634

[5] Ladhani S Bhutta ZA Neonatal Pseudomonas putida infection presenting as staphylococcal scalded skin syndrome. Eur J Clin Microbiol Infect Dis 1998;17:642–4. 9832266 10.1007/BF01708347

[6] Martino R Martinez C Pericas R Bacteremia due to glucose non-fermenting gram-negative bacilli in patients with hematological neoplasias and solid tumors. Eur J Clin Microbiol Infect Dis 1996;15:610–5. 8874083 10.1007/BF01709374

[7] Lombardi G Luzzaro F Docquier JD Nosocomial infections caused by multidrug-resistant isolates of Pseudomonas putida producing VIM-1 metallo-beta-lactamase. J Clin Microbiol 2002;40:4051–5. 12409373 10.1128/JCM.40.11.4051-4055.2002 PMC139695

[8] Cho CH Lee SB Comparison of clinical characteristics and antibiotic susceptibility between Pseudomonas aeruginosa and P. putida keratitis at a tertiary referral center: a retrospective study. BMC Ophthalmol 2018;18:204. 30126384 10.1186/s12886-018-0882-3 PMC6102849

[9] Taylor M Keane CT Falkiner FR Pseudomonas putida in transfused blood. Lancet 1984;2:107. 10.1016/s0140-6736(84)90279-4 6145996

[10] Anaissie E Fainstein V Miller P Pseudomonas putida. Newly recognized pathogen in patients with cancer. Am J Med 1987;82:1191–4. 3605136 10.1016/0002-9343(87)90223-3

[11] Yoshino Y Kitazawa T Kamimura M Pseudomonas putida bacteremia in adult patients: five case reports and a review of the literature. J Infect Chemother 2011;17:278–82. 20809240 10.1007/s10156-010-0114-0

[12] Yang CH Young T Peng MY Clinical spectrum of Pseudomonas putida infection. J Formos Med Assoc 1996;95:754–61. 8961672

[13] Thomas BS Okamoto K Bankowski MJ A lethal case of Pseudomonas putida bacteremia due to soft tissue infection. Infect Dis Clin Pract (Baltim Md) 2013;21:147–213. 23750097 10.1097/IPC.0b013e318276956b PMC3673730

[14] Roig P Orti A Navarro V Meningitis due to Pseudomonas stutzeri in a patient infected with human immunodeficiency virus. Clin Infect Dis 1996;22:587–8. 8852995 10.1093/clinids/22.3.587

[15] Loiseau-Marolleau ML Malarre N [Pseudomonas putida: identification, antibiotic sensitivity and pathogenicity (author's transl)]. Pathol Biol (Paris) 1977;25:637–45. 341053

[16] Molina L Udaondo Z Duque E Specific gene loci of clinical Pseudomonas putida isolates. PLoS One 2016;11:e147478. 10.1371/journal.pone.0147478 PMC4731212 26820467

[17] Bouallegue O Mzoughi R Weill FX Outbreak of Pseudomonas putida bacteraemia in a neonatal intensive care unit. J Hosp Infect 2004;57:88–91. 15142722 10.1016/j.jhin.2004.01.024

[18] Franzetti F Cernuschi M Esposito R Pseudomonas infections in patients with AIDS and AIDS-related complex. J Intern Med 1992;231:437–43. 1588272 10.1111/j.1365-2796.1992.tb00957.x

[19] Kim SE Park SH Park HB Nosocomial Pseudomonas putida bacteremia: high rates of carbapenem resistance and mortality. Chonnam Med J 2012;48:91–5. 22977749 10.4068/cmj.2012.48.2.91 PMC3434797

[20] Molina L Udaondo Z Duque E Antibiotic resistance determinants in a Pseudomonas putida strain isolated from a hospital. PLoS One 2014;9:e81604. 24465371 10.1371/journal.pone.0081604 PMC3894933

[21] Sader HS Jones RN Antimicrobial susceptibility of uncommonly isolated non-enteric Gram-negative bacilli. Int J Antimicrob Agents 2005;25:95–109. 15664479 10.1016/j.ijantimicag.2004.10.002

[22] Baran W Adamek E Ziemianska J Effects of the presence of sulfonamides in the environment and their influence on human health. J Hazard Mater 2011;196:1–5. 21955662 10.1016/j.jhazmat.2011.08.082

[23] Jacquier H Carbonnelle E Corvec S Revisited distribution of nonfermenting Gram-negative bacilli clinical isolates. Eur J Clin Microbiol Infect Dis 2011;30:1579–86. 21509476 10.1007/s10096-011-1263-5

